# Integrated consensus genetic and physical maps of flax (*Linum usitatissimum* L.)

**DOI:** 10.1007/s00122-012-1953-0

**Published:** 2012-08-14

**Authors:** Sylvie Cloutier, Raja Ragupathy, Evelyn Miranda, Natasa Radovanovic, Elsa Reimer, Andrzej Walichnowski, Kerry Ward, Gordon Rowland, Scott Duguid, Mitali Banik

**Affiliations:** 1 Cereal Research Centre, Agriculture and Agri-Food Canada, 195 Dafoe Road, Winnipeg, MB R3T 2M9 Canada; 2 Department of Plant Science, University of Manitoba, 66 Dafoe Road, Winnipeg, MB R3T 2N2 Canada; 3 Crop Development Centre, University of Saskatchewan, 51 Campus Drive, Saskatoon, SK S7N 5A8 Canada; 4 Morden Research Station, Agriculture and Agri-Food Canada, 101 Route 100, Unit 100, Morden, MB R6M 1Y5 Canada

## Abstract

Three linkage maps of flax (*Linum usitatissimum* L.) were constructed from populations CDC Bethune/Macbeth, E1747/Viking and SP2047/UGG5-5 containing between 385 and 469 mapped markers each. The first consensus map of flax was constructed incorporating 770 markers based on 371 shared markers including 114 that were shared by all three populations and 257 shared between any two populations. The 15 linkage group map corresponds to the haploid number of chromosomes of this species. The marker order of the consensus map was largely collinear in all three individual maps but a few local inversions and marker rearrangements spanning short intervals were observed. Segregation distortion was present in all linkage groups which contained 1–52 markers displaying non-Mendelian segregation. The total length of the consensus genetic map is 1,551 cM with a mean marker density of 2.0 cM. A total of 670 markers were anchored to 204 of the 416 fingerprinted contigs of the physical map corresponding to ~274 Mb or 74 % of the estimated flax genome size of 370 Mb. This high resolution consensus map will be a resource for comparative genomics, genome organization, evolution studies and anchoring of the whole genome shotgun sequence.

**Electronic supplementary material:**

The online version of this article (doi:10.1007/s00122-012-1953-0) contains supplementary material, which is available to authorized users.

## Introduction

Flax (*Linum usitatissimum* L., 2*n* = 2*x* = 30), is an annual self-pollinated crop that is commercially grown as a source of stem fibre and seed oil. Flax seed oil is utilized for the fabrication of various biodegradable products such as high quality drying oil, paints, varnishes and linoleum flooring. Flax oil is a rich source of omega-3 fatty acids used as nutraceuticals and also as a functional food for both humans and animals. Fibre and oilseed flax belong to the same species but are morphologically different. Oilseed type flax plants (linseed) are more branched and shorter than the fibre type (Gill 1987). Fibre flax is grown mainly in Northern Europe, Russia and China but linseed is the primary type grown in Canada, USA, Argentina and India as well as Russia and China (Gill 1987; Marchenkov et al. 2003).

Development and characterization of flax genetic resources and assessment of genetic variability are essential for germplasm conservation and breeding. Flax germplasm collections contain thousands of accessions of *L. usitatissimum* and related species, of which, subsets were assessed for the extent of genetic diversity for morphological characteristics (Diederichsen and Hammer 1995; Diederichsen 2001; Diederichsen and Raney 2006; Saeidi 2008). A variety of molecular markers including random amplified polymorphic DNA (RAPD), restriction fragment length polymorphism (RFLP), amplified fragment length polymorphism (AFLP) and simple sequence repeat (SSR) have been developed and used in assessing flax genetic diversity (Spielmeyer et al. 1998; Oh et al. 2000; Wiesner et al. 2001; Fu et al. 2003; Adugna et al. 2006; Fu 2006, Roose-Amsaleg et al. 2006; Cloutier et al. 2009, 2012; Uysal et al. 2010; Deng et al. 2010, 2011; Bickel et al. 2011; Kale et al. 2012; Rachinskaya et al. 2011; Soto-Cerda et al. 2011a, 2011b). While the reports are numerous, the number of informative markers in each of the studies is somewhat limited with the majority reporting between 9 and 60 markers only (Cloutier et al. 2012).

SSRs are stretches of DNA consisting of a variable number of short tandem repeats that are generally co-dominant, highly polymorphic, multi-allelic, relatively abundant, heritable, reproducible and reliable (Powell et al. 1996; Hwang et al. 2009). They also show cross-species usefulness and can be used in closely related species (Powell et al. 1996; Collard et al. 2005; Varshney et al. 2005). SSRs have been developed from genomic sequences or Expressed Sequence Tags (ESTs). In flax, Ragupathy et al. (2011) identified 4,064 putative SSRs from bacterial artificial chromosome (BAC) end sequences (BES). SSR markers have also been developed from various flax EST libraries (Cloutier et al. 2009; Soto-Cerda et al. 2011b) and from SSR-enriched genomic libraries or other genomic sequences (Roose-Amsaleg et al. 2006; Deng et al. 2010, 2011; Bickel et al. 2011; Kale et al. 2012; Rachinskaya et al. 2011). There are currently 1,326 SSR markers published in flax (Cloutier et al. 2012). SSR markers have been used for the construction of genetic maps of many plant species and provide dependable landmarks throughout the genome (Cheng et al. 2009; Studer et al. 2010). In flax, genetic maps and genetic diversity assessment were achieved with this type of marker (Fu and Peterson 2010; Cloutier et al. 2011; Soto-Cerda et al. 2011b). Genetic maps are useful for evolutionary and comparative studies as they provide both intra- and inter-species genome wide insights on recombination rates and gene rearrangements within or across chromosomes (Ball et al. 2010; Wang et al. 2011).

Only three individual flax linkage maps (Spielmeyer et al. 1998; Oh et al. 2000; Cloutier et al. 2011) have been published to date. The linkage map developed by Cloutier et al. (2011) had 113 markers, mostly SSRs, grouped into 24 linkage groups, while those of Spielmeyer et al. (1998) and Oh et al. (2000) were based on 213 AFLP markers forming 18 linkage groups and 94 RFLP/RAPD markers grouped into 15 linkage groups, respectively. The limitations of these maps reside in either or both the type and limited number of markers. Hence the need exists for a reliable, high density genetic map of flax that would serve as reference for a wide variety of applications such as QTL mapping, map based gene cloning, marker assisted crop improvement, linkage disequilibrium (LD) mapping, phylogenetic analysis and anchoring of the whole genome shotgun sequence assembly. Consensus genetic linkage maps have been constructed for various plant species including *Arabidopsis* (Hauge et al. 1993), *Brassica* (Xu et al. 2010, Wang et al. 2011), barley (Langridge et al. 1995; Varshney et al. 2007), sorghum (Mace et al. 2009), wheat (Somers et al. 2004), rice (Antonio et al. 1996), maize (Cone et al. 2002), red clover (Isobe et al. 2009), lettuce (Truco et al. 2007), rye (Gustafson et al. 2009), soybean (Hwang et al. 2009), melon (Diaz et al. 2011), grapevine (Vezzulli et al. 2008), cowpea (Muchero et al. 2009), chickpea (Millan et al. 2010), potato (Danan et al. 2011), eucalyptus (Brondani et al. 2006), *Cucurbita pepo* (Zraidi et al. 2007) and *Zoysia* species (Li et al. 2010). High density consensus maps are well suited as references for the incorporation of information from genetically diverse individuals or multiple populations thus facilitating comparative analyses across germplasm.

Whole genome physical maps have been constructed for maize (Messing et al. 2004), *Brachypodium* (Gu et al. 2009), melon (González et al. 2010), grapevine (Scalabrin et al. 2010), *Arabidopsis* (Mozo et al. 1999), *Brassica rapa* (Mun et al. 2008), soybean (Wu et al. 2004), apple (Han et al. 2011) and flax (Ragupathy et al. 2011). BAC-based physical maps have been anchored to genetic maps in a number of plants such as rice (Chen et al. 2002), maize (Wei et al. 2009), papaya (Yu et al. 2009), *Medicago* (Mun et al. 2006), bean (Córdoba et al. 2010), poplar (Kelleher et al. 2007), grapevine (Scalabrin et al. 2010) and melon (González et al. 2010), where they were used to order physical maps and provide a framework for genome sequence assemblies.

The physical map of the flax genome cv. CDC Bethune, an oilseed flax variety, consists of 416 fingerprinted contigs (FPC) spanning ~368 Mb, very close to the estimated genome size of CDC Bethune of approximately 370 Mb (Ragupathy et al. 2011). The present study was intended to: (1) construct three independent genetic maps, (2) create an integrated consensus genetic map and (3) anchor the consensus genetic and physical maps of flax to provide the backbone information for ordering the whole genome shotgun (WGS) sequence assembly.

## Materials and methods

### Plant material, DNA extraction and marker amplification

Three segregating populations were used for mapping. CDC Bethune/Macbeth (BM) is comprised of 243 F_6_-derived recombinant inbred lines (RILs). The two parents are current varieties termed ‘conventional’ oilseed types because they contain 55–57 % linolenic acid, a “standard” amount for oilseed flax varieties. E1747/Viking (EV) received from S. Knapp (University of Georgia, USA) consists of 90 F_6_-derived RILs generated from a cross between the low linolenic acid line E1747 and the European fibre flax variety Viking. SP2047/UGG5-5 (SU) is an F_1_-derived doubled haploid (DH) population of 78 individuals. SP2047 is a solin breeding line characterized by its 2–4 % linolenic acid content and yellow seeds while UGG5-5 is a “high-lin” line with 65–70 % linolenic acid (Banik et al. 2011; Cloutier et al. 2011).

Genomic DNA was extracted from lyophilized leaf tissue (~100 mg fresh) of individual seedlings of all the segregating and parental lines of the three mapping populations using the DNeasy 96 plant kit according to manufacturer’s instructions (Qiagen Inc, Toronto, ON, Canada). The genomic DNA was quantified by fluorometer and re-suspended to a final concentration of 6 ng/μl. Amplification of template DNA with SSR primers was performed in 384–well plates in a final volume of 10 μl. The amplification products were resolved on an ABI 3130xl Genetic analyzer (Applied Biosystems, Foster City, CA, USA) and scored for segregation of parental alleles at each SSR locus. A total of five SNPs and seven genes (*fad2A*, *fad2B*, *fad3A*, *fad3B*, *dgatA*, *dgatB and ysc1*) were also positioned on the maps (Cloutier et al. 2011). Protocols and primer information for SSR markers Lu4 to Lu1193, Lu2097 to Lu2300 and Lu2331 to Lu3291 were previously described (Cloutier et al. 2009, 2011, 2012). In addition, SSR markers Lu2001 to Lu2096 were from previously published reports (Roose-Amsaleg et al. 2006; Deng et al. 2010, 2011) while Lu2301 to Lu2330 were designed from scaffold 505 of the flax WGS sequence assembly (http://www.phytozome.net), as previously described (Cloutier et al. 2009, 2012). References for individual markers of the consensus map are listed (Supplementary Table S1).

### Anchoring the genetic and physical maps

Genetic and physical map anchoring was performed using complementary strategies. First, the CDC Bethune BAC library was screened with a subset of the SSR primers positioned on the genetic maps to identify BAC addresses and their corresponding FPC contigs (Ragupathy et al. 2011). SSR markers Lu2097-Lu2300 and Lu2331-Lu3291 were derived directly from BESs and, as such, were directly assigned BAC addresses and corresponding FPC contigs. These BES anchors were confirmed by performing BLASTn searches of the SSR primer sequences against the flax WGS sequence assembly (http://www.phytozome.net). Only perfect matches of both primer sequences to the same scaffold were considered for anchoring. BLASTn searches were also performed using the BESs from which the SSRs were derived and matches with an *e* value <1e-25 were considered scaffold anchors. Similarly, primer BLAST and BLASTn were performed for SSR markers Lu4 to Lu1193 derived from ESTs (Cloutier et al. 2009, 2012). The marker name convention “marker name_FPC contig number” (e.g. Lu3156_405) was adopted to indicate positioning of the markers on the physical map. Markers not anchored to an FPC contig were labelled ‘0’ (e.g. Lu2312_0). Markers amplifying two or three loci were labelled with a single contig number but likely belong to paralogous contigs.

### Map construction and linkage analysis

Linkage analysis was carried out independently for each mapping population using JoinMap 4.0 (Van Ooijen 2006) with a LOD of 4.0 and a maximum recombination frequency of 40. Marker segregation was tested against the expected Mendelian ratio of 1:1 using the Chi-square goodness-of-fit test. For conversion of recombination frequency into map distances expressed in centiMorgans (cM), the Kosambi mapping function which accounts for genetic interference from double cross over was used (Kosambi 1944).

The consensus linkage map was constructed based on the principle illustrated by Stam (1993) using JoinMap 4.0 (Van Ooijen 2006). LOD scores and pairwise recombination frequencies were computed for all linkage groups (LGs) of individual populations. They were then combined into a single group node in the navigation tree using the ‘grouping node’ command. Consensus LGs were obtained using the ‘combine groups for map integration’ function that is based on the presence of a minimum of two marker loci common to at least two populations on the basis of the mean recombination frequencies and combined LOD scores of pairwise data from the three segregating populations. The consensus map was constructed according to Alheit et al. (2011) and Gong et al. (2008) using the following parameters: Kosambi mapping function, regression mapping option, maximum recombination frequency of 40, LOD >1.0, ripple = 1, third round = yes and goodness-of-fit jump threshold for removal of loci = 5.0. Comparative analyses of marker distance and marker order were performed across individual maps and with the consensus map by visualization of the four final maps obtained as described above. The individual homologous LGs from the three populations were integrated using commonly mapped markers based on 411 segregating lines (243 RILs from BM, 90 RILs from EV and 78 DHs from SU). The 15 consensus LGs were numbered LG1 to LG15, in decreasing size order (cM). The population specific LGs were described using the population acronyms and the same number as in the consensus map (BMLG1-BMLG16, EVLG1-EVLG18 and SULG1-SULG15).

## Results

### Individual genetic linkage maps

#### CDC Bethune/Macbeth

A total of 389 segregating marker loci were polymorphic in the BM population, of which, 385 marker loci assembled into 16 LGs spanning 2,007 cM, for an average density of one marker locus every 5.2 cM, and 4 marker loci remained unlinked (Table 1). LGs ranged from 27 to 187 cM and contained 9–40 markers. The *dgatA* and *dgatB* genes were mapped in this population. The population showed segregation distortion for 56 loci (*P* < 0.05) with equal numbers of loci skewed towards each parent (Supplementary Table S2).

**Table 1:** Mapping statistics for the three individual and the consensus genetic maps of flax

Populations	No. individuals	Total no. marker loci	No. marker loci in LGs^a^	Length (cM)	No. LGs^a^	No. unlinked marker loci	No. marker loci in the consensus map	Percent marker loci in the consensus map
CDC Bethune/Macbeth (BM)	243	389	385	2,007	16	4	373	95.9
E1747/Viking (EV)	90	443	442	1,731	18	1	419	94.6
SP2047/UGG5-5 (SU)	78	477	469	3,044	15	8	463	97.1
Consensus	411	821	770 (19)	1,551	15 (4)	32	770	93.8

^a^Numbers in brackets represent marker loci and LGs belonging to a single population and that did not incorporate in the 15 LGs of the consensus map

#### E1747/Viking

The EV population was assayed with 443 polymorphic marker loci, 442 of which grouped into 18 LGs leaving a single marker unlinked (Table 1). The length of the LGs and number of marker loci per LG varied from 10 to 168 cM and 2–46, respectively. The total length of the map was 1,731 cM with a mean marker density of 3.9 cM between loci. 77 of the 442 loci diverged significantly from the expected 1:1 segregation ratio with 33 skewed towards E1747 and 44 skewed towards Viking (Supplementary Table S2).

#### SP2047/UGG5-5

The previously published SU map was based on 125 marker loci assembled in 24 linkage groups spanning 834 cM (Cloutier et al. 2011). The SU map constructed herein is more saturated and comprehensive with 477 polymorphic marker loci, of which 8 remained unlinked and the remaining 469 formed 15 LGs totalling 3,044 cM (Table 1). The length of the LGs and the number of loci per LG varied from 136 to 362 cM and 20–47, respectively. The approximate average marker density was one every 6.5 cM. A total of 168 loci showed segregation distortion (Supplementary Table S2). All LGs contained distorted markers except SULG13 which contained a single polymorphic marker in the SU population and SULG14 (Supplementary Table S2). A total of seven genes including six from fatty acid biosynthetic pathways (*fad2A*, *fad2B*, *fad3A*, *fad3B*, *dgatA* and *dgatB*) were also mapped.

### Consensus map

**Fig. 1:**
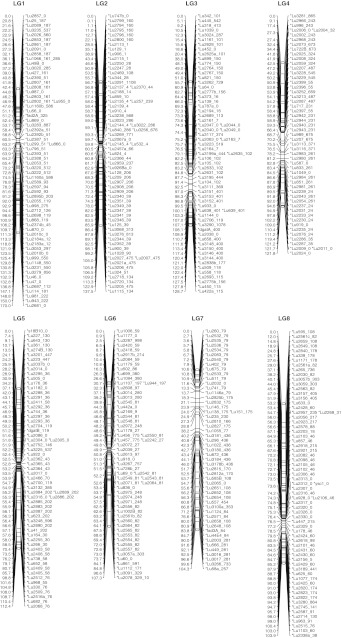

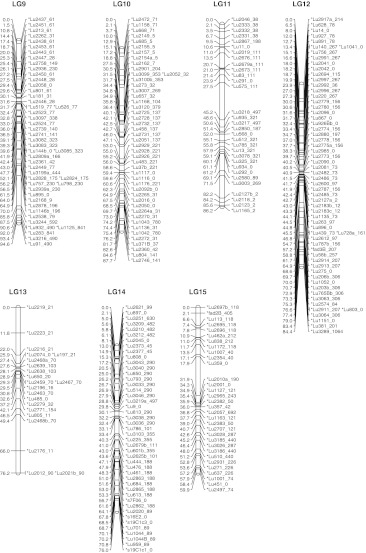
Consensus genetic map of flax integrated from three mapping populations. Numbers to the *left* of each linkage group represent Kosambi map units (cM). Locus names followed by their FPC contig anchor separated by an underscore are on the *right*. Linkage groups are in decreasing size order

A total of 795 markers generated 821 loci for a locus per marker ratio of 1.03 because 18 markers identified two loci and four markers identified three loci. Of the 821 loci scored in the three populations, 114 were common to all three populations and another 257 were common to two of the three populations (Supplementary Table S3). A total of 770 marker loci were assembled into the 15 LGs constituting the consensus map (Fig. 1; Table 1). Four additional LGs contained 19 marker loci ordered based on a single mapping population and 32 markers remained unlinked (Table 1; Supplementary Table S2). The total length of the consensus genetic linkage map was 1,551 cM and LGs ranged from 60 to 170 cM. The consensus map had an average marker density of one per 2.0 cM. Assuming a genome size of 370 Mb for CDC Bethune (Ragupathy et al. 2011), the genome wide ratio of physical to genetic distance averaged 239 Kb/cM, equivalent to an average of one marker per 481 Kb.

The length of the consensus map was shorter than the individual population specific maps (Table 1). All LGs of the consensus map were constructed based on markers shared among the three populations except LG13 which was constructed mostly with markers from the BM and EV populations because all markers, with the exception of Lu850, were monomorphic in the SU population. The consensus map displayed a few gaps that were mostly smaller than 10 cM. The largest gap of 20.8 cM is in LG1 (Fig. 1; Supplementary Table S1). The marker orders were consistent between the three independent genetic maps with some local inversions.

The percentages of distorted loci in BM, EV and SU populations were 15, 17 and 36 %, respectively (Supplementary Table S2). Out of the 770 loci of the consensus map, 292 (38 %) loci exhibited segregation distortion in at least one of the three mapping populations (Supplementary Table S2). The presence of a large number of distorted loci seems to have caused the large gap in LG11. Although LG8 carried the most distorted loci (76 %), the length of that LG was not affected as compared to the bi-parental populations where marker segregation was Mendelian, although some local rearrangements of closely linked markers were observed. Overall, only a few ambiguities were identified with respect to marker position compared to individual maps. Markers Lu359_0 and Lu2354_40, common to all three populations, mapped at the proximal end of each individual population LG15. However, these markers were placed at internal positions (41.977 and 42.829 cM) in the consensus LG15 (Supplementary Table S1).

### Anchoring genetic and physical maps

**Fig. 2:**
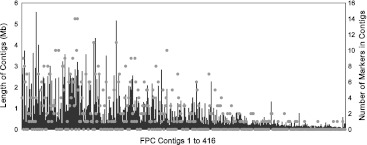
Distribution of the genetic markers of the consensus map across the FPC contigs of the physical map (Ragupathy et al. 2011). A total of 204 of the 416 FPC contigs (*x* axis) were anchored by 670 marker loci (*dots*). The length of the contigs (Mb) is on the left *y* axis. From 1 to 14 marker loci (*dots*) were anchored onto each FPC contig represented on the right *y* axis

Of the 770 loci in the consensus genetic map, 670 were anchored to 204 of the 416 FPC contigs of the physical map (Ragupathy et al. 2011) corresponding to 274 Mb or 74 % of the flax genome (Fig. 2). Twenty-one of the 204 FPC contigs were anchored at more than one map location because some markers amplified two or three polymorphic loci (labelled with a small a, b or c suffix in Fig. 1). FPC contigs were anchored with 1–14 markers. Examples of FPC contigs anchored with 14 markers include FPC contigs 79 and 82 estimated at 2.836 Mb with 205 BAC clones and 3.192 Mb with 265 BAC clones, respectively. The largest FPC contig (21), estimated at 5.562 Mb, consisted of 437 BAC clones and was anchored with four markers. Sixty-seven FPC contigs contained a single marker.

## Discussion

### Comparison of individual and consensus maps

Three genetic maps of flax have been published to date: a 213 AFLP marker-based map of 18 LGs covering 1,400 cM (Spielmeyer et al. 1998), a 1,000 cM RFLP/RAPD map with 94 markers assembled into 15 LGs (Oh et al. 2000) and a 113 marker map containing EST-SSRs, SNPs, genes and one phenotypic trait grouped into 24 LGs and spanning 834 cM (Cloutier et al. 2011). Major QTL for *fusarium* wilt (Spielmeyer et al. 1998), for fatty acid composition and seed coat colour (Cloutier et al. 2011) were identified using these genetic maps. The increased marker density of the SU map from 113 (Cloutier et al. 2011) to 469 (this publication) significantly improved the map by bridging gaps and thereby reducing the number of LGs from 24 to 15 while increasing the map coverage from 834 to 3,044 cM which promises to enhance QTL detection. Collinearity with two additional genetic maps (BM and EV) further confirmed the accuracy of the groupings. The three maps were largely collinear with few marker inversions. Although some LGs or portions thereof were fixed in some of the individual populations (e.g. LG4, LG13 and LG15 in SU; LG7, LG9 and LG15 in BM; LG15 in EV), the consensus map successfully bridged LGs and resulted in good coverage across the genome with few gaps. Some local inconsistencies of marker order such as small inversions or local rearrangements between individual and consensus maps were observed, particularly in closely linked markers and markers located at the distal ends of LGs as previously reported for rye (Studer et al. 2010), cotton (Xu et al. 2008), *Zoysia* (Li et al. 2010), grapevine (Vezzulli et al. 2008) and *Eucalyptus* (Brondani et al. 2006). The single most striking discrepancy resided in the total length of the SU map which exceeded the size of the BM and EV maps by more than 1,000 cM. The higher percentage (36 %) of distorted markers of this DH population, i.e. at least twice as high as the other two populations, may be responsible for the artifactual expansion of the genetic map length (Garcia-Dorado and Gallego 1992; Zhu et al. 2007; Li et al. 2011).

Comparative and consensus mapping are advantageous to obtain an unbiased linkage map representing the genome under investigation. As discussed above, mapping of markers employing multiple populations provides increased genome coverage because it is unlikely that multiple parents would all be fixed (monomorphic) in the same genomic regions. Also, overall population size afforded by multiple populations increases the chances of capturing recombination events, the foundation of genetic mapping. Reports of overrepresentation of localized crossover promoting 13mer motifs (Myers et al. 2008) in recombination hot spots of 1–2 kb (Ptak et al. 2005), and the influence of highly polymorphic trans-acting loci such as PRDM9 on the activation of those recombination hotspots in human (Baudat et al. 2010; Paigen and Petkov 2010) indicates the importance of the genomic background in crossover frequencies. Considering crossing over as a fundamental cellular process conserved across eukaryotes, variability for distribution of recombination hot spots and its genetic determinants can be determined using multiple populations. Comparative mapping can also offer evidence for duplications or chromosomal rearrangements (Sewell et al. 1999). As a consequence of merging of datasets from three populations, the consensus map had fewer and smaller gaps compared to the individual genetic maps, hence it was more comprehensive. Fatty acid desaturase genes *fad2A*, *fad2B*, *fad3A* and *fad3B*, diacyl glycerol transferase genes *dgatA* and *dgatB* and seed coat color gene *ysc1* were positioned to seven different LGs of the consensus map. The majority were polymorphic in a single population but common neighbouring polymorphic markers permitted their integration in the consensus map, illustrating another advantage of consensus mapping. Flax has a relatively low level of genetic polymorphism, indicating a lower degree of genome divergence (Deng et al. 2010; Cloutier et al. 2011; Kale et al. 2012), unlike crops like maize where extensive molecular variation has been reported, primarily due to the activity of transposable elements (Llaca et al. 2011). The use of multiple populations followed by consensus mapping greatly increases marker saturation, a valuable feature for all mapping applications, for understanding the LD structure across genomes and germplasm characterization by association mapping (Soto-Cerda and Cloutier 2012).

The present consensus map of 770 SSR markers represents a major improvement over the low resolution phylogenetic analyses published to date with other marker types (McDill et al. 2009; Fu and Allaby 2010) and those published with few SSR markers within *Linum usitatissimum* (Wiesner et al. 2001; Cloutier et al. 2009) and across *Linum* species (Fu and Peterson 2010; Soto-Cerda et al. 2011b). Pale flax (*Linum bienne* Mill, *L. angustifolia* Huds) is the wild progenitor of cultivated flax. Both have similar karyotypes bearing equal numbers of chromosomes (2n = 2x = 30) (Muravenko et al. 2003) and interspecific crosses between them produce fertile progeny (Gill and Yermanos 1967; Diederichsen and Hammer 1995). Pale and cultivated flax have been inferred to differ by a single translocation event (Gill and Yermanos 1967). The exceptionally high transferability (97 %) of EST-SSRs from cultivated flax to *L. bienne* supports the assignment of pale flax to the primary gene pool (Diederichsen 2007; Fu and Peterson 2010; Soto-Cerda et al. 2011b). A genetic map for pale flax or an interspecific cross map has yet to be produced. The current availability of SSR markers combined with their cross applicability should allow for an in-depth analysis of genetic diversity in *L. bienne* which should be useful to explore its potential to widen the gene pool of cultivated flax to meet breeding objectives.

*Linum usitatissimum* is a self-pollinated diploid species which, like a number of crop genomes, is an ancient polyploid (Blanc and Wolfe 2004; Paterson et al. 2004; Pfeil et al. 2005; Sterck et al. 2005; Gong et al. 2008; Soltis et al. 2009; Schmutz et al. 2010; Jiao et al. 2011; Lin and Paterson 2011). The remnant of ancestral whole genome duplication is reflected by the fact that a subset of SSR markers amplified two paralogous loci, although in most cases, only one was polymorphic (Cloutier et al. 2009, 2012). Mapping of the markers that amplified multiple polymorphic loci revealed ancestral chromosomal rearrangements resulting from paleopolyploidization events as noticed in LG6 and LG8. The existence of duplicated regions in consensus linkage groups LG6 and LG8 delimited by Lu2561 and Lu3057 markers indicate signatures of ancient duplication (Supplementary Figure 1). Analyses of a large collection of flax ESTs also corroborate the ancient duplication of flax (Venglat et al. 2011), as exemplified by the duplicate nature of the genes of the fatty acid biosynthetic pathway (Cloutier et al. 2011). Global comparative analysis of the scaffolds of the WGS sequence assembly promises a more comprehensive picture of the events that have shaped the flax genome through evolution.

### Distorted markers

The segregation distortion in the three populations ranged from 15 to 36 %, an intermediate level, comparable to extent of distorted markers reported in common bean (37.3 %, de Campos et al. 2011), maize (19–36 %, Lu et al. 2002), red clover (5.8–45 %, Isobe et al. 2009), *Medicago truncatula* (27 %, Thoquet et al. 2002) and peanut (8.5–22.8 %, Hong et al. 2010) but higher than *C. pepo* (3.7 %, Zraidi et al. 2007; Gong et al. 2008), *Brassica rapa* (2.6 %, Song et al. 1991), grapevine (7–11 %, Doligez et al. 2006) and globe artichoke (13 %, Portis et al. 2009). Species such as *Arabidopsis* (43.0 %, Reiter et al. 1992), cotton (71 %, Lacape et al. 2009), tomato (68 %, Paterson et al. 1988), perennial ryegrass (32–63 %, Anhalt et al. 2008), *Zoysia* (43.7 %, Li et al. 2010) and cowpea (41 %, Muchero et al. 2009), all displayed substantially higher percentages of markers deviating from the expected segregation ratios.

Non-Mendelian segregation ratios arise from chromosomal rearrangements, gametic competition, embryo viability and various physiological causes (Xu et al. 1997; Gonzalo et al. 2005; Portis et al. 2009) and, inadvertently, also from sampling errors (Lorieux et al. 2000). Segregation distortion has been associated more strongly with genetic effects as opposed to population structure or marker type (Anhalt et al. 2008).

Among the three flax populations reported here, the DH SU population had the highest proportion of distorted loci, similar to DH populations of rice (31.8 %, Xu et al. 1997) and *Brassica* (22–49 %, Wang et al. 2011), but lower than alfalfa (68 %, Li et al. 2011). The higher proportion of non-Mendelian markers in DH populations may be attributed to selection for tissue culture responsiveness loci (Xu et al. 1997; Alheit et al. 2011). Even though they were located on all LGs, distorted markers were not randomly distributed but were clustered within LGs (Cloutier et al. 2011; Córdoba et al. 2010; Li et al. 2011) supporting the cause of selection rather than experimental errors (Li et al. 2011), a view further emphasized by higher proportions of distorted markers on specific LGs such as LG2 (36/65), LG8 (52/68) and LG10 (32/46). Chromosome specific uneven distribution of markers has been reported for triticale chromosome 2A and 1R (Alheit et al. 2011); LG1, LG2 and LG3 of *Medicago truncatula* (Studer et al. 2010) and M20 and M32 of *Zoysia* (Li et al. 2010). Clustering of distorted markers was also documented in lettuce (Truco et al. 2007), *Eucalyptus* (Brondani et al. 2006) and peanut (Hong et al. 2010).

Map distance and map order can both be affected by segregation distortion (Lyttle 1991; Zhu et al. 2007) as was observed in the SU population where large gaps were observed between blocks of non-Mendelian markers adjacent to blocks of markers with non-skewed segregation, which accounted in part for the overestimation of the map length for this population. Elimination of non-Mendelian marker loci was suggested to improve mapping accuracies (Zhu et al. 2007; Xu 2008). However, such an approach would decrease the number of markers available and reduce coverage of some genomic regions, hence diminishing the map saturation (Zhu et al. 2007; Xu 2008). Here, we clearly demonstrated that consensus mapping was a powerful way to correct for mapping inaccuracies caused by non-Mendelian markers because consensus mapping takes into account segregation data from multiple populations including common markers with Mendelian segregation in at least one population.

### Anchoring genetic and physical maps

The physical map of flax is comprised of 416 FPC contigs spanning ~368 Mb (Ragupathy et al. 2011). A total of 670 markers were anchored to 204 FPC contigs representing ~274 Mb, i.e. 74 % of the estimated 370 Mb genome of CDC Bethune, comparable to papaya (72.4 %, Yu et al. 2009), apple (60 %, Han et al. 2011) and grapevine (72 %, Scalabrin et al. 2010) genomes and exceeding the extent of anchoring reported in *Medicago truncatula* (32 %, Mun et al. 2006), *Populus trichocarpa* (22 %, Kelleher et al. 2007), *Prunus* (15.5 %, Zhebentyayeva et al. 2008), bean (8 %, Córdoba et al. 2010) and melon (12 %, González et al. 2010). Although sufficient to provide initial ordering of the WGS sequence assembly into bins, the level of anchoring of the physical and genetic maps of flax presented herein falls short of the requirement for high accuracy ordering and orienting of the scaffolds of genomic sequence as was shown in maize (93 %, Wei et al. 2009) and rice (91 %, Chen et al. 2002). Tens of thousands of genome-wide SNPs currently being developed in our lab from the three mapping populations, using the state of the art ‘genotyping by sequencing (GBS)’ approach (Davey et al. 2011) will likely provide the degree of saturation necessary for the task of obtaining an accurate physical map ordering and orientation, a prerequisite for a high quality genome sequence assembly.

In conclusion, we reported on the construction of the first consensus genetic map of flax using 411 individuals from three populations and grouping and ordering 770 markers in 15 LGs spanning 1,551 cM. The vast majority of the markers are SSRs, a highly reproducible marker system which should prove its usefulness as an important resource for the flax research community, especially flax breeders. The overall map density averaged one marker every 2.0 cM. The consensus genetic map has been anchored to the flax physical map, a first step in the ordering of the scaffolds that currently make up the WGS sequence assembly of the flax genome. This integrated map will enable structural and functional genomic studies including fine mapping of genes of interest, marker-assisted flax breeding, map-based gene cloning, comparative/synteny mapping, QTL analysis and association mapping in flax and other related species.

## Electronic supplementary material

Below is the link to the electronic supplementary material.

Supplementary material 1 (PDF 91 kb)

Supplementary material 2 (PDF 446 kb)

Supplementary material 3 (PDF 38 kb)

Supplementary material 4 (PDF 15 kb)
