# Supplementary figures and images for: Integrated consensus genetic and physical maps of flax (Linum usitatissimum L.)
Source: Theor Appl Genet. 2012 Aug 14;125(8):1783–95. doi: 10.1007/s00122-012-1953-0 (PMC3493668; doi:10.1007/s00122-012-1953-0)

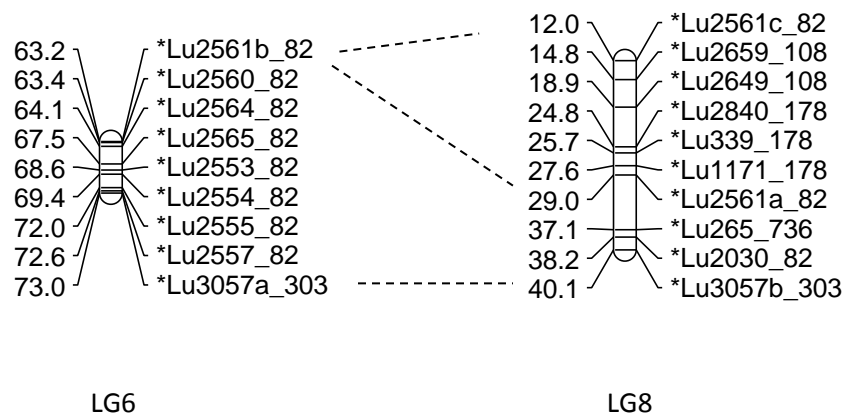

Fig S1: Segment of the two consensus linkage groups LG6 and LG8 representing a duplicated region.

Supplement: Supplementary file 1 — Supplementary material 1 (PDF 91 kb) [file 122_2012_1953_MOESM1_ESM.pdf]
