# Supplementary material for: Integrated consensus genetic and physical maps of flax (Linum usitatissimum L.)
Source: Theor Appl Genet. 2012 Aug 14;125(8):1783–95. doi: 10.1007/s00122-012-1953-0 (PMC3493668; doi:10.1007/s00122-012-1953-0)
Supplement: Supplementary file 2 — Supplementary material 2 (PDF 446 kb) [file 122_2012_1953_MOESM2_ESM.pdf]

Supplementary Data Table S1: Description of the consensus genetic map of flax. Loci, their position and reference are listed for each linkage group. Distorted markers are identified with and asterisk (*).

Linkage Group	S/n	Locus	Position	Reference
LG1	1	Lu2857_187*	0	Cloutier et al. (2012)
LG1	2	Lu25_187	20.83	Cloutier et al. (2009)
LG1	3	Lu2089_187*	21.643	Roose-Amsaleg et al. (2006)
LG1	4	Lu3235_537*	22.182	Cloutier et al. (2012)
LG1	5	Lu2026_560	22.86	Deng et al. (2011)
LG1	6	Lu2853_187	24.517	Cloutier et al. (2012)
LG1	7	Lu2861_187	26.597	Cloutier et al. (2012)
LG1	8	Lu2091_0*	27.316	Roose-Amsaleg et al. (2006)
LG1	9	Lu2858_187	32.115	Cloutier et al. (2012)
LG1	10	Lu1066_161_285	36.106	Cloutier et al. (2012)
LG1	11	Lu49B_0	36.6	Cloutier et al. (2009)
LG1	12	Lu3020_285	37.156	Cloutier et al. (2012)
LG1	13	Lu427_161*	38.966	Cloutier et al. (2009)
LG1	14	Lu2390_51*	40.768	Cloutier et al. (2012)
LG1	15	Lu2807_161*	41.795	Cloutier et al. (2012)
LG1	16	Lu2808_161*	42.351	Cloutier et al. (2012)
LG1	17	Lu987_0*	44.844	Cloutier et al. (2012)
LG1	18	Lu2803_161	46.16	Cloutier et al. (2012)
LG1	19	Lu2802_161*	46.863	Cloutier et al. (2012)
LG1	20	Lu955_0*	46.87	Cloutier et al. (2012)
LG1	21	Lu1160b_586*	47.626	Cloutier et al. (2012)
LG1	22	Lu56_0	51.059	Cloutier et al. (2009)
LG1	23	fad2A_325*	51.209	Cloutier et al. (2011)
LG1	24	Lu869_0	52.362	Cloutier et al. (2012)
LG1	25	Lu3283_887	53.722	Cloutier et al. (2012)
LG1	26	Lu2392a_51	58.367	Cloutier et al. (2012)
LG1	27	Lu2392b_51	58.806	Cloutier et al. (2012)
LG1	28	Lu2387_51	60.498	Cloutier et al. (2012)
LG1	29	Lu299_51	63.178	Cloutier et al. (2009)
LG1	30	Lu866_0	63.2	Cloutier et al. (2012)
LG1	31	Lu796_51	63.297	Cloutier et al. (2009)
LG1	32	Lu2393_51	63.923	Cloutier et al. (2012)
LG1	33	Lu2388_51	64.215	Cloutier et al. (2012)
LG1	34	Lu2053_51	65.403	Deng et al. (2010)
LG1	35	Lu3220_512	70.189	Cloutier et al. (2012)
LG1	36	Lu3222_512	72.815	Cloutier et al. (2012)
LG1	37	Lu1160a_586	74.505	Cloutier et al. (2012)
LG1	38	Lu2589_93	76.822	Cloutier et al. (2012)
LG1	39	Lu2597_94*	78.281	Cloutier et al. (2012)
LG1	40	Lu2592_93	81.605	Cloutier et al. (2012)
LG1	41	Lu2895b_203	91.193	Cloutier et al. (2012)
LG1	42	Lu2055_119	91.97	Deng et al. (2010)
LG1	43	Lu998_275	92.917	Cloutier et al. (2012)

LG1	44	Lu2712_126	94.433	Cloutier et al. (2012)
LG1	45	Lu2698_119	98.568	Cloutier et al. (2012)
LG1	46	Lu868_119	100.857	Cloutier et al. (2012)
LG1	47	Lu2374b_45	106.188	Cloutier et al. (2012)
LG1	48	Lu870_0	109.745	Cloutier et al. (2012)
LG1	49	Lu2010c_0	111.332	Deng et al. (2011)
LG1	50	Lu2184a_12	113.326	Cloutier et al. (2012)
LG1	51	Lu2183a_12	118.77	Cloutier et al. (2012)
LG1	52	Lu3053_297	121.942	Cloutier et al. (2012)
LG1	53	Lu2010b_0*	123.471	Deng et al. (2011)
LG1	54	Lu999_550*	125.391	Cloutier et al. (2012)
LG1	55	Lu1148_550	129.829	Cloutier et al. (2012)
LG1	56	Lu3231_550*	130.763	Cloutier et al. (2012)
LG1	57	Lu3279_856	131.258	Cloutier et al. (2012)
LG1	58	Lu46_0*	132.272	Cloutier et al. (2009)
LG1	59	Lu47_0	132.764	Cloutier et al. (2009)
LG1	60	Lu2687_112*	136.798	Cloutier et al. (2012)
LG1	61	Lu114_181	147.076	Cloutier et al. (2009)
LG1	62	Lu981_222*	148.832	Cloutier et al. (2012)
LG1	63	Lu943_222*	149.996	Cloutier et al. (2012)
LG1	64	Lu2681_0*	170	Cloutier et al. (2012)

	S/n	Locus	Position	
LG2	1	Lu747b_160	0	Cloutier et al. (2009)
LG2	2	Lu2799_160	0.107	Cloutier et al. (2012)
LG2	3	Lu2794_160	1.07	Cloutier et al. (2012)
LG2	4	Lu2795_160	10.987	Cloutier et al. (2012)
LG2	5	Lu2796_160	12.691	Cloutier et al. (2012)
LG2	6	Lu2800_160*	22.071	Cloutier et al. (2012)
LG2	7	Lu2113_1*	28.521	Cloutier et al. (2012)
LG2	8	Lu129_1	35.104	Cloutier et al. (2009)
LG2	9	Lu906_1*	38.891	Cloutier et al. (2012)
LG2	10	Lu2115_1	43.384	Cloutier et al. (2012)
LG2	11	Lu2250_28*	46.599	Cloutier et al. (2012)
LG2	12	Lu2247_28	50.876	Cloutier et al. (2012)
LG2	13	Lu2469_108*	53.466	Cloutier et al. (2012)
LG2	14	Lu344_28	55.656	Cloutier et al. (2009)
LG2	15	Lu3291_1108	56.706	Cloutier et al. (2012)
LG2	16	Lu2137_4	56.75	Cloutier et al. (2012)
LG2	17	Lu2370_44*	56.829	Cloutier et al. (2012)
LG2	18	Lu2188_14*	57.113	Cloutier et al. (2012)
LG2	19	Lu859_10	57.38	Cloutier et al. (2012)
LG2	20	Lu2135_4	57.987	Cloutier et al. (2012)
LG2	21	Lu257_239*	58.033	Cloutier et al. (2009)
LG2	22	Lu2139_4*	58.398	Cloutier et al. (2012)
LG2	23	Lu910_4*	58.683	Cloutier et al. (2012)
LG2	24	Lu3238_568*	58.874	Cloutier et al. (2012)

LG2	25	Lu3023_286*	59.047	Cloutier et al. (2012)
LG2	26	Lu2144_4*	59.053	Cloutier et al. (2012)
LG2	27	Lu3022_286*	59.057	Cloutier et al. (2012)
LG2	28	Lu840_286	59.095	Cloutier et al. (2009)
LG2	29	Lu3256_676*	59.104	Cloutier et al. (2012)
LG2	30	Lu3269_771*	59.301	Cloutier et al. (2012)
LG2	31	Lu2067a_0	59.724	Deng et al. (2010)
LG2	32	Lu2145_4*	59.819	Cloutier et al. (2012)
LG2	33	Lu532_4	59.832	Cloutier et al. (2009)
LG2	34	Lu2457a_66	60.461	Cloutier et al. (2012)
LG2	35	Lu824_4	60.613	Cloutier et al. (2009)
LG2	36	Lu2366_44	60.804	Cloutier et al. (2012)
LG2	37	Lu2959_237*	61.714	Cloutier et al. (2012)
LG2	38	Lu926Ba_0*	62.86	Cloutier et al. (2012)
LG2	39	Lu128_206	67.021	Cloutier et al. (2009)
LG2	40	Lu209_206*	68.281	Cloutier et al. (2009)
LG2	41	Lu2907_206*	70.022	Cloutier et al. (2012)
LG2	42	Lu2908_206*	70.5	Cloutier et al. (2012)
LG2	43	Lu2909_206	71.28	Cloutier et al. (2012)
LG2	44	Lu2340_39	72.416	Cloutier et al. (2012)
LG2	45	Lu2351_39*	74.272	Cloutier et al. (2012)
LG2	46	Lu2349_39	78.007	Cloutier et al. (2012)
LG2	47	Lu2347_39*	81.968	Cloutier et al. (2012)
LG2	48	Lu2341_39*	82.478	Cloutier et al. (2012)
LG2	49	Lu2346_39*	83.542	Cloutier et al. (2012)
LG2	50	Lu125_39*	85.882	Cloutier et al. (2009)
LG2	51	Lu3068_313	86.355	Cloutier et al. (2012)
LG2	52	Lu3276_813	87.789	Cloutier et al. (2012)
LG2	53	Lu2344_39*	89.144	Cloutier et al. (2012)
LG2	54	Lu2352_39	92.526	Cloutier et al. (2012)
LG2	55	Lu900_39	98.126	Cloutier et al. (2012)
LG2	56	Lu1028_90	99.198	Cloutier et al. (2012)
LG2	57	Lu2027_475*	105.012	Deng et al. (2011)
LG2	58	Lu2007_475*	105.021	Deng et al. (2011)
LG2	59	Lu2021a_475*	105.021	Deng et al. (2011)
LG2	60	Lu3206_475*	105.828	Cloutier et al. (2012)
LG2	61	Lu324_51*	106.528	Cloutier et al. (2009)
LG2	62	Lu2718_134*	109.194	Cloutier et al. (2012)
LG2	63	Lu2720_134*	113.259	Cloutier et al. (2012)
LG2	64	Lu3205_475*	122.899	Cloutier et al. (2012)
LG2	65	Lu1115_134	137.549	Cloutier et al. (2012)

	S/n	Locus	Position	
LG3	1	Lu342_101	0	Cloutier et al. (2009)
LG3	2	Lu445_542*	5.113	Cloutier et al. (2009)
LG3	3	Lu318_413*	9.172	Cloutier et al. (2009)
LG3	4	Lu1039_0*	10.466	Cloutier et al. (2012)

LG3	5	Lu3024_287	12.292	Cloutier et al. (2012)
LG3	6	Lu1161_101*	15.401	Cloutier et al. (2012)
LG3	7	Lu2628_101*	15.596	Cloutier et al. (2012)
LG3	8	Lu452_0	18.617	Cloutier et al. (2009)
LG3	9	Lu2625a_101*	20.357	Cloutier et al. (2012)
LG3	10	Lu899_150	38.029	Cloutier et al. (2012)
LG3	11	Lu774_150	48.082	Cloutier et al. (2009)
LG3	12	Lu2764_150	48.97	Cloutier et al. (2012)
LG3	13	Lu2767_150	49.429	Cloutier et al. (2012)
LG3	14	Lu821_150	51.006	Cloutier et al. (2009)
LG3	15	Lu3262_729	55.74	Cloutier et al. (2012)
LG3	16	Lu64_0	60.881	Cloutier et al. (2009)
LG3	17	Lu2777b_156*	62.678	Cloutier et al. (2012)
LG3	18	Lu373_16	64.449	Cloutier et al. (2009)
LG3	19	Lu139_16	65.998	Cloutier et al. (2009)
LG3	20	Lu787a_0	69.315	Cloutier et al. (2009)
LG3	21	Lu2194_16	72.308	Cloutier et al. (2012)
LG3	22	Lu2689_113	73.265	Cloutier et al. (2012)
LG3	23	Lu2161_7*	73.576	Cloutier et al. (2012)
LG3	24	Lu2047_7*	74.278	Deng et al. (2010)
LG3	25	Lu2044_7*	74.328	Deng et al. (2010)
LG3	26	Lu2040_7*	74.361	Deng et al. (2010)
LG3	27	Lu2049_7	74.375	Deng et al. (2010)
LG3	28	Lu3117_374	74.437	Cloutier et al. (2012)
LG3	29	Lu2063_0	75.199	Deng et al. (2010)
LG3	30	Lu2163_7	75.21	Cloutier et al. (2012)
LG3	31	Lu3223_519*	75.655	Cloutier et al. (2012)
LG3	32	Lu2164_7	76.492	Cloutier et al. (2012)
LG3	33	Lu3199a_444*	79.09	Cloutier et al. (2012)
LG3	34	Lu2635_102*	79.117	Cloutier et al. (2012)
LG3	35	Lu106_102	82.174	Cloutier et al. (2009)
LG3	36	Lu105_102*	82.48	Cloutier et al. (2009)
LG3	37	Lu2633_102	83.228	Cloutier et al. (2012)
LG3	38	Lu2631_102*	84.791	Cloutier et al. (2012)
LG3	39	Lu3195_444*	87.7	Cloutier et al. (2012)
LG3	40	Lu104_102	91.402	Cloutier et al. (2009)
LG3	41	Lu3111_369	92.547	Cloutier et al. (2012)
LG3	42	Lu3151_401	97.042	Cloutier et al. (2012)
LG3	43	Lu3153_401	98.339	Cloutier et al. (2012)
LG3	44	Lu3152_401	98.841	Cloutier et al. (2012)
LG3	45	Lu933_0	99.492	Cloutier et al. (2012)
LG3	46	Lu638_401	100.086	Cloutier et al. (2009)
LG3	47	Lu639_401	100.101	Cloutier et al. (2009)
LG3	48	Lu1144_0	103.279	Cloutier et al. (2012)
LG3	49	Lu2706_119	107.026	Cloutier et al. (2012)
LG3	50	Lu3290_1078	107.116	Cloutier et al. (2012)
LG3	51	dgatA_400	108.064	Cloutier et al. (2011)

LG3	52	Lu2038_0	108.918	Deng et al. (2011)
LG3	53	Lu658_400	110.721	Cloutier et al. (2009)
LG3	54	Lu3148_400	111.624	Cloutier et al. (2012)
LG3	55	Lu3150_400	113.379	Cloutier et al. (2012)
LG3	56	Lu3146_400	117.373	Cloutier et al. (2012)
LG3	57	Lu3144_400	120.397	Cloutier et al. (2012)
LG3	58	Lu2838b_177	121.06	Cloutier et al. (2012)
LG3	59	Lu509_118	121.952	Cloutier et al. (2009)
LG3	60	Lu558_118	122.399	Cloutier et al. (2009)
LG3	61	Lu2693_115	127.443	Cloutier et al. (2012)
LG3	62	Lu2775b_156	127.488	Cloutier et al. (2012)
LG3	63	Lu450_115	127.765	Cloutier et al. (2009)
LG3	64	Lu422a_115*	128.672	Cloutier et al. (2009)

	S/n	Locus	Position	
LG4	1	Lu3281_865*	0	Cloutier et al. (2012)
LG4	2	Lu2966_243	9.144	Cloutier et al. (2012)
LG4	3	Lu996_243	17.448	Cloutier et al. (2012)
LG4	4	Lu2006_0*	27.305	Deng et al. (2011)
LG4	5	Lu2004_320	27.315	Deng et al. (2011)
LG4	6	Lu2002_243	27.325	Deng et al. (2011)
LG4	7	Lu2968_243	27.372	Cloutier et al. (2012)
LG4	8	Lu2073_673	32.371	Deng et al. (2010)
LG4	9	Lu722B_673*	34.416	Cloutier et al. (2009)
LG4	10	Lu2025_324	41.841	Deng et al. (2011)
LG4	11	Lu2008_324	41.874	Deng et al. (2011)
LG4	12	Lu2059_324	42.501	Deng et al. (2010)
LG4	13	Lu2207_487	47.168	Cloutier et al. (2012)
LG4	14	Lu3228_545*	49.183	Cloutier et al. (2012)
LG4	15	Lu3229_545*	50.209	Cloutier et al. (2012)
LG4	16	Lu2399_55	50.878	Cloutier et al. (2012)
LG4	17	Lu2396_55*	53.787	Cloutier et al. (2012)
LG4	18	Lu3252_659*	53.889	Cloutier et al. (2012)
LG4	19	Lu3213_487*	54.096	Cloutier et al. (2012)
LG4	20	Lu2087_487*	54.171	Roose-Amsaleg et al. (2006)
LG4	21	Lu717_231*	57.794	Cloutier et al. (2009)
LG4	22	Lu2397_55	58.37	Cloutier et al. (2012)
LG4	23	Lu2942_231*	58.802	Cloutier et al. (2012)
LG4	24	Lu2944_231	58.865	Cloutier et al. (2012)
LG4	25	Lu2940_231*	59.273	Cloutier et al. (2012)
LG4	26	Lu2943_231	66.387	Cloutier et al. (2012)
LG4	27	Lu989_675	68.716	Cloutier et al. (2012)
LG4	28	Lu207_675	70.184	Cloutier et al. (2009)
LG4	29	Lu3113_371	75.485	Cloutier et al. (2012)
LG4	30	Lu3116_371	76.478	Cloutier et al. (2012)
LG4	31	Lu2983_261*	78.156	Cloutier et al. (2012)
LG4	32	Lu2980_261	81.181	Cloutier et al. (2012)

LG4	33	Lu587_0*	82.811	Cloutier et al. (2009)
LG4	34	Lu833_261	83.426	Cloutier et al. (2009)
LG4	35	Lu1049_0*	84.524	Cloutier et al. (2012)
LG4	36	Lu2984_261	86.586	Cloutier et al. (2012)
LG4	37	Lu851_261*	87.827	Cloutier et al. (2009)
LG4	38	Lu2981_261*	88.865	Cloutier et al. (2012)
LG4	39	Lu2239_24	89.515	Cloutier et al. (2012)
LG4	40	Lu2043_261	91.761	Deng et al. (2010)
LG4	41	Lu2054_261*	92.033	Deng et al. (2010)
LG4	42	Lu2237_24	94.552	Cloutier et al. (2012)
LG4	43	Lu2031_24*	94.86	Deng et al. (2011)
LG4	44	Lu2233_24	97.805	Cloutier et al. (2012)
LG4	45	Lu2230_24*	102.198	Cloutier et al. (2012)
LG4	46	Lu919_0	108.987	Cloutier et al. (2012)
LG4	47	Lu2235_24	110.389	Cloutier et al. (2012)
LG4	48	Lu2076_24	111.246	Roose-Amsaleg et al. (2006)
LG4	49	Lu2286_35	119.356	Cloutier et al. (2012)
LG4	50	Lu2287_35	121.222	Cloutier et al. (2012)
LG4	51	Lu2009_0	121.738	Deng et al. (2011)
LG4	52	Lu2011_0	121.738	Deng et al. (2011)
LG4	53	Lu2024_0	121.768	Deng et al. (2011)

	S/n	Locus	Position	
LG5	1	s18B10_0	0	Cloutier et al. (2011)
LG5	2	Lu227_130	7.353	Cloutier et al. (2009)
LG5	3	Lu643_130	9.132	Cloutier et al. (2009)
LG5	4	Lu361_130	10.733	Cloutier et al. (2009)
LG5	5	Lu274B_130	13.542	Cloutier et al. (2009)
LG5	6	Lu3201_447	24.059	Cloutier et al. (2012)
LG5	7	Lu223_447	24.244	Cloutier et al. (2009)
LG5	8	Lu2037b_0*	25.944	Deng et al. (2011)
LG5	9	Lu2014_0*	28.23	Deng et al. (2011)
LG5	10	Lu2295_36	28.875	Cloutier et al. (2012)
LG5	11	Lu505_0	30.934	Cloutier et al. (2009)
LG5	12	Lu176_36	34.235	Cloutier et al. (2009)
LG5	13	Lu1182_0	37.117	Cloutier et al. (2012)
LG5	14	Lu2288_36	41.959	Cloutier et al. (2012)
LG5	15	Lu2291_36	43.099	Cloutier et al. (2012)
LG5	16	Lu2411_58	44.051	Cloutier et al. (2012)
LG5	17	Lu2292_36	45.694	Cloutier et al. (2012)
LG5	18	Lu744_36	46.931	Cloutier et al. (2009)
LG5	19	Lu2297_36	48.258	Cloutier et al. (2012)
LG5	20	Lu2293_36	49.326	Cloutier et al. (2012)
LG5	21	Lu2704_119	50.191	Cloutier et al. (2012)
LG5	22	dgatB_119	51.173	Cloutier et al. (2011)
LG5	23	Lu2086_0	51.599	Roose-Amsaleg et al. (2006)
LG5	24	Lu2304_0	51.789	www.phytozome.net/Linum usitatissimum/Scaffold505

LG5	25	Lu2305_0	51.789	www.phytozome.net/Linum usitatissimum/Scaffold505
LG5	26	Lu2752_146	51.928	Cloutier et al. (2012)
LG5	27	Lu3225_537	52.212	Cloutier et al. (2012)
LG5	28	Lu922_0	52.39	Cloutier et al. (2012)
LG5	29	Lu2362_43	52.54	Cloutier et al. (2012)
LG5	30	Lu2365_43	52.782	Cloutier et al. (2012)
LG5	31	Lu2364_43	53.191	Cloutier et al. (2012)
LG5	32	Lu2017_0	53.467	Deng et al. (2011)
LG5	33	Lu2466_70	53.872	Cloutier et al. (2012)
LG5	34	Lu2700_119	54.668	Cloutier et al. (2012)
LG5	35	Lu3132_385	54.943	Cloutier et al. (2012)
LG5	36	Lu2884_202	55.214	Cloutier et al. (2012)
LG5	37	Lu2889_202	55.214	Cloutier et al. (2012)
LG5	38	Lu2318_0	55.271	www.phytozome.net/Linum usitatissimum/Scaffold505
LG5	39	Lu2886_202	55.31	Cloutier et al. (2012)
LG5	40	Lu2885_202	55.428	Cloutier et al. (2012)
LG5	41	Lu2883_202	55.865	Cloutier et al. (2012)
LG5	42	Lu2887_202	56.122	Cloutier et al. (2012)
LG5	43	Lu623_202	56.278	Cloutier et al. (2009)
LG5	44	Lu3248_596	56.409	Cloutier et al. (2012)
LG5	45	Lu2890_202	56.605	Cloutier et al. (2012)
LG5	46	Lu41_202	57.082	Cloutier et al. (2009)
LG5	47	Lu164_30	57.614	Cloutier et al. (2009)
LG5	48	Lu2255_30	57.995	Cloutier et al. (2012)
LG5	49	Lu266_30	58.475	Cloutier et al. (2009)
LG5	50	Lu2403_58	70.368	Cloutier et al. (2012)
LG5	51	Lu2408_58	73.518	Cloutier et al. (2012)
LG5	52	Lu738_58	76.169	Cloutier et al. (2009)
LG5	53	Lu652_58	79.072	Cloutier et al. (2009)
LG5	54	Lu2409_58*	80.71	Cloutier et al. (2012)
LG5	55	Lu2405_58	85.68	Cloutier et al. (2012)
LG5	56	Lu2512_76	98.82	Cloutier et al. (2012)
LG5	57	Lu968_55	101.626	Cloutier et al. (2012)
LG5	58	Lu330_76	104.773	Cloutier et al. (2009)
LG5	59	Lu2509_76	107.964	Cloutier et al. (2012)
LG5	60	Lu2516a_76	108.706	Cloutier et al. (2012)
LG5	61	Lu682_76	110.369	Cloutier et al. (2009)
LG5	62	Lu2068_76	112.44	Deng et al. (2010)

	S/n	Locus	Position	
LG6	1	Lu1006_59	0	Cloutier et al. (2012)
LG6	2	Lu1177_0	0.941	Cloutier et al. (2012)
LG6	3	Lu3287_998	2.451	Cloutier et al. (2012)
LG6	4	Lu2420_59	6.485	Cloutier et al. (2012)
LG6	5	Lu2418_59	8.654	Cloutier et al. (2012)
LG6	6	Lu2917b_214	9.603	Cloutier et al. (2012)
LG6	7	Lu2084_59	10.357	Roose-Amsaleg et al. (2006)

LG6	8	Lu1179_59	10.808	Cloutier et al. (2012)
LG6	9	Lu502_86	16.267	Cloutier et al. (2009)
LG6	10	Lu699_380	26.287	Cloutier et al. (2009)
LG6	11	Lu1094_380	26.64	Cloutier et al. (2012)
LG6	12	Lu1107_197*	30.718	Cloutier et al. (2012)
LG6	13	Lu944_197*	30.731	Cloutier et al. (2012)
LG6	14	Lu2608_97	32.31	Cloutier et al. (2012)
LG6	15	Lu3014_280	37.144	Cloutier et al. (2012)
LG6	16	Lu3013_280	39.15	Cloutier et al. (2012)
LG6	17	Lu2545_81*	39.795	Cloutier et al. (2012)
LG6	18	Lu442a_0	44.304	Cloutier et al. (2009)
LG6	19	Lu2169_9*	45.815	Cloutier et al. (2012)
LG6	20	Lu2544_81*	46.581	Cloutier et al. (2012)
LG6	21	Lu2548_81*	48.102	Cloutier et al. (2012)
LG6	22	Lu2972_248*	48.243	Cloutier et al. (2012)
LG6	23	Lu1178_27*	48.594	Cloutier et al. (2012)
LG6	24	Lu456_775*	49.21	Cloutier et al. (2009)
LG6	25	Lu2550_81*	49.234	Cloutier et al. (2012)
LG6	26	Lu457_775*	49.404	Cloutier et al. (2009)
LG6	27	Lu2242_27	49.422	Cloutier et al. (2012)
LG6	28	Lu2072_27*	49.819	Deng et al. (2010)
LG6	29	Lu2039_27*	49.881	Deng et al. (2010)
LG6	30	Lu2613_97*	50.819	Cloutier et al. (2012)
LG6	31	Lu918_0	51.031	Cloutier et al. (2012)
LG6	32	Lu3267_757*	51.307	Cloutier et al. (2012)
LG6	33	Lu728b_27	51.53	Cloutier et al. (2009)
LG6	34	Lu69_0	52.207	Cloutier et al. (2009)
LG6	35	Lu2542_81	52.207	Cloutier et al. (2012)
LG6	36	Lu2549_81	52.207	Cloutier et al. (2012)
LG6	37	Lu2543_81	52.227	Cloutier et al. (2012)
LG6	38	Lu2071_81*	52.578	Deng et al. (2010)
LG6	39	Lu2064_81*	52.578	Deng et al. (2010)
LG6	40	Lu836_0	53.386	Cloutier et al. (2009)
LG6	41	Lu2975_248*	53.684	Cloutier et al. (2012)
LG6	42	Lu2974_248*	54.85	Cloutier et al. (2012)
LG6	43	Lu2971_248*	57.53	Cloutier et al. (2012)
LG6	44	Lu2556_82	62.5	Cloutier et al. (2012)
LG6	45	Lu1002B_82	62.64	Cloutier et al. (2012)
LG6	46	Lu2561b_82	63.175	Cloutier et al. (2012)
LG6	47	Lu2560_82	63.358	Cloutier et al. (2012)
LG6	48	Lu2564_82	64.098	Cloutier et al. (2012)
LG6	49	Lu2565_82	67.521	Cloutier et al. (2012)
LG6	50	Lu2553_82	68.561	Cloutier et al. (2012)
LG6	51	Lu2554_82*	69.379	Cloutier et al. (2012)
LG6	52	Lu2555_82*	72.007	Cloutier et al. (2012)
LG6	53	Lu2557_82	72.636	Cloutier et al. (2012)
LG6	54	Lu3057a_303*	72.997	Cloutier et al. (2012)

LG6	55	Lu60_0	73.741	Cloutier et al. (2009)
LG6	56	Lu861_591	84.096	Cloutier et al. (2012)
LG6	57	Lu1112_171	84.842	Cloutier et al. (2012)
LG6	58	Lu3091_329*	96.565	Cloutier et al. (2012)
LG6	59	Lu2078_329_10	107.304	Roose-Amsaleg et al. (2006)

	S/n	Locus	Position	
LG7	1	Lu260_79	0	Cloutier et al. (2009)
LG7	2	Lu2532_79	2.731	Cloutier et al. (2012)
LG7	3	Lu2535_79	3.589	Cloutier et al. (2012)
LG7	4	Lu2536_79	5.652	Cloutier et al. (2012)
LG7	5	Lu2534_79	5.794	Cloutier et al. (2012)
LG7	6	Lu2083_79	6.718	Roose-Amsaleg et al. (2006)
LG7	7	Lu2540_79	7.207	Cloutier et al. (2012)
LG7	8	Lu402_79	7.942	Cloutier et al. (2009)
LG7	9	Lu675_79	10.646	Cloutier et al. (2009)
LG7	10	Lu2533_79	10.838	Cloutier et al. (2012)
LG7	11	Lu511_79	10.852	Cloutier et al. (2009)
LG7	12	Lu2032_0	11.575	Deng et al. (2011)
LG7	13	Lu741_79	13.335	Cloutier et al. (2009)
LG7	14	Lu1146a_79	13.742	Cloutier et al. (2012)
LG7	15	Lu2825b_175	18.291	Cloutier et al. (2012)
LG7	16	Lu2832_175	21.083	Cloutier et al. (2012)
LG7	17	Lu146_175	23.949	Cloutier et al. (2009)
LG7	18	Lu138_175	23.982	Cloutier et al. (2009)
LG7	19	Lu151_175	23.997	Cloutier et al. (2009)
LG7	20	Lu235_230	24.941	Cloutier et al. (2009)
LG7	21	Lu2810_166	27.289	Cloutier et al. (2012)
LG7	22	Lu2827_175	28.785	Cloutier et al. (2012)
LG7	23	Lu1055_77	38.221	Cloutier et al. (2012)
LG7	24	Lu3181_436	40.196	Cloutier et al. (2012)
LG7	25	Lu296_436	40.777	Cloutier et al. (2009)
LG7	26	Lu1022_436	42.295	Cloutier et al. (2012)
LG7	27	Lu3180_436	42.655	Cloutier et al. (2012)
LG7	28	Lu672_436	43.414	Cloutier et al. (2009)
LG7	29	Lu3184_436	44.13	Cloutier et al. (2012)
LG7	30	Lu3178b_436	46.046	Cloutier et al. (2012)
LG7	31	Lu2815_170	51.728	Cloutier et al. (2012)
LG7	32	Lu2812a_170	52.473	Cloutier et al. (2012)
LG7	33	Lu585B_108*	53.671	Cloutier et al. (2009)
LG7	34	Lu2065_0	54.696	Deng et al. (2010)
LG7	35	Lu2651_108	55.553	Cloutier et al. (2012)
LG7	36	Lu2652_108	56.151	Cloutier et al. (2012)
LG7	37	Lu2654_108*	56.257	Cloutier et al. (2012)
LG7	38	Lu557_454*	60.775	Cloutier et al. (2009)
LG7	39	Lu3100a_353*	66.767	Cloutier et al. (2012)
LG7	40	Lu1124_84*	71.157	Cloutier et al. (2012)

LG7	41	Lu2571_84	76.588	Cloutier et al. (2012)
LG7	42	Lu2658_108*	79.993	Cloutier et al. (2012)
LG7	43	Lu2648_108*	80.903	Cloutier et al. (2012)
LG7	44	fad3A_84	83.801	Cloutier et al. (2011)
LG7	45	Lu44E4_84*	84.702	Cloutier et al. (2011)
LG7	46	Lu2003_281*	90.004	Deng et al. (2011)
LG7	47	Lu566_281*	91.551	Cloutier et al. (2009)
LG7	48	Lu449_281	92.09	Cloutier et al. (2009)
LG7	49	Lu3016_281	92.541	Cloutier et al. (2012)
LG7	50	Lu3017_281	96.588	Cloutier et al. (2012)
LG7	51	Lu3266_733	99.588	Cloutier et al. (2012)
LG7	52	Lu58a_257	104.327	Cloutier et al. (2009)

	S/n	Locus	Position	
LG8	1	Lu595_108*	0	Cloutier et al. (2009)
LG8	2	Lu2561c_82*	11.97	Cloutier et al. (2012)
LG8	3	Lu2659_108*	14.847	Cloutier et al. (2012)
LG8	4	Lu2649_108*	18.926	Cloutier et al. (2012)
LG8	5	Lu2840_178*	24.789	Cloutier et al. (2012)
LG8	6	Lu339_178*	25.67	Cloutier et al. (2009)
LG8	7	Lu1171_178*	27.623	Cloutier et al. (2012)
LG8	8	Lu2561a_82	28.956	Cloutier et al. (2012)
LG8	9	Lu265_736	37.142	Cloutier et al. (2009)
LG8	10	Lu2030_82*	38.213	Deng et al. (2011)
LG8	11	Lu3057b_303*	40.082	Cloutier et al. (2012)
LG8	12	Lu3059_303*	44.045	Cloutier et al. (2012)
LG8	13	Lu2563_82*	45.388	Cloutier et al. (2012)
LG8	14	Lu3157_405*	47.867	Cloutier et al. (2012)
LG8	15	Lu3156_405*	51.939	Cloutier et al. (2012)
LG8	16	Lu633_0	53.165	Cloutier et al. (2009)
LG8	17	Lu2428_60*	53.412	Cloutier et al. (2012)
LG8	18	Lu2957_235*	54.695	Cloutier et al. (2012)
LG8	19	Lu2268_31*	54.697	Cloutier et al. (2012)
LG8	20	Lu2056_217*	55.961	Deng et al. (2010)
LG8	21	Lu2923_217*	56.328	Cloutier et al. (2012)
LG8	22	Lu2578_88*	56.608	Cloutier et al. (2012)
LG8	23	Lu2203_18*	57.063	Cloutier et al. (2012)
LG8	24	Lu2103_46	64.941	Cloutier et al. (2012)
LG8	25	Lu857_46*	65.302	Cloutier et al. (2012)
LG8	26	Lu2918_215*	66.13	Cloutier et al. (2012)
LG8	27	Lu2921_215*	67.561	Cloutier et al. (2012)
LG8	28	Lu2082_46*	70.199	Roose-Amsaleg et al. (2006)
LG8	29	Lu2098_46*	70.806	Cloutier et al. (2012)
LG8	30	Lu2105_46*	72.129	Cloutier et al. (2012)
LG8	31	Lu2102_46*	72.661	Cloutier et al. (2012)
LG8	32	Lu2306_46	73.014	www.phytozome.net/Linum usitatissimum/Scaffold505
LG8	33	Lu2313_0	73.502	www.phytozome.net/Linum usitatissimum/Scaffold505

LG8	34	Lu2312_0*	73.626	www.phytozome.net/Linum usitatissimum/Scaffold505
LG8	35	ysc1_0*	73.628	Cloutier et al. (2011)
LG8	36	Lu2307_0*	73.628	www.phytozome.net/Linum usitatissimum/Scaffold505
LG8	37	Lu2316_46	74.167	www.phytozome.net/Linum usitatissimum/Scaffold505
LG8	38	Lu928_0*	74.273	Cloutier et al. (2012)
LG8	39	Lu2106_46	74.296	Cloutier et al. (2012)
LG8	40	Lu2317_0	74.757	www.phytozome.net/Linum usitatissimum/Scaffold505
LG8	41	Lu2320_0*	75.118	www.phytozome.net/Linum usitatissimum/Scaffold505
LG8	42	Lu2326_0*	75.288	www.phytozome.net/Linum usitatissimum/Scaffold505
LG8	43	Lu2330_0*	76.368	www.phytozome.net/Linum usitatissimum/Scaffold505
LG8	44	Lu447_215*	76.494	Cloutier et al. (2009)
LG8	45	Lu2329_0*	77.153	www.phytozome.net/Linum usitatissimum/Scaffold505
LG8	46	Lu178_46*	78.509	Cloutier et al. (2009)
LG8	47	Lu2424_60*	79.839	Cloutier et al. (2012)
LG8	48	Lu2618_98*	80.798	Cloutier et al. (2012)
LG8	49	Lu2101_46	83.497	Cloutier et al. (2012)
LG8	50	Lu2431_60	84.461	Cloutier et al. (2012)
LG8	51	Lu2430_60*	84.568	Cloutier et al. (2012)
LG8	52	Lu2156_5	84.745	Cloutier et al. (2012)
LG8	53	Lu2429_60*	85.205	Cloutier et al. (2012)
LG8	54	Lu3189_441	87.9	Cloutier et al. (2012)
LG8	55	Lu625_60*	88.652	Cloutier et al. (2009)
LG8	56	Lu1077_174*	88.775	Cloutier et al. (2012)
LG8	57	Lu2425_60*	89.994	Cloutier et al. (2012)
LG8	58	Lu2820_174	91.01	Cloutier et al. (2012)
LG8	59	Lu2823_174*	92.959	Cloutier et al. (2012)
LG8	60	Lu2822_174*	93.81	Cloutier et al. (2012)
LG8	61	Lu3280_864*	94.211	Cloutier et al. (2012)
LG8	62	Lu2745_141	94.729	Cloutier et al. (2012)
LG8	63	Lu2587_91*	95.689	Cloutier et al. (2012)
LG8	64	Lu2714_130*	98	Cloutier et al. (2012)
LG8	65	Lu963_91*	99.018	Cloutier et al. (2012)
LG8	66	Lu2515_76	100.366	Cloutier et al. (2012)
LG8	67	Lu1103_60*	103.025	Cloutier et al. (2012)
LG8	68	Lu2338a_38*	103.929	Cloutier et al. (2012)

	S/n	Locus	Position	
LG9	1	Lu2437_61	0	Cloutier et al. (2012)
LG9	2	Lu2451_61	1.459	Cloutier et al. (2012)
LG9	3	Lu213_61	10.845	Cloutier et al. (2009)
LG9	4	Lu2262_31*	14.43	Cloutier et al. (2012)
LG9	5	Lu2438_61	17.361	Cloutier et al. (2012)
LG9	6	Lu2453_61	18.737	Cloutier et al. (2012)
LG9	7	Lu2443_61*	19.995	Cloutier et al. (2012)
LG9	8	Lu2447_28*	25.309	Cloutier et al. (2012)
LG9	9	Lu2758_149	25.391	Cloutier et al. (2012)
LG9	10	Lu2936_230	26.119	Cloutier et al. (2012)

LG9	11	Lu2450_61	27.215	Cloutier et al. (2012)
LG9	12	Lu2448_28*	27.485	Cloutier et al. (2012)
LG9	13	Lu2058_0	29.399	Deng et al. (2010)
LG9	14	Lu801_61*	29.614	Cloutier et al. (2009)
LG9	15	Lu181_31*	30.146	Cloutier et al. (2009)
LG9	16	Lu2446_28*	31.623	Cloutier et al. (2012)
LG9	17	Lu519_77	31.66	Cloutier et al. (2009)
LG9	18	Lu526_77	31.712	Cloutier et al. (2009)
LG9	19	Lu2523_77	32.428	Cloutier et al. (2012)
LG9	20	Lu3097_338*	32.764	Cloutier et al. (2012)
LG9	21	Lu2524_77	33.006	Cloutier et al. (2012)
LG9	22	Lu2739_140*	35.974	Cloutier et al. (2012)
LG9	23	Lu2741_141	36.525	Cloutier et al. (2012)
LG9	24	Lu3082_323	39.13	Cloutier et al. (2012)
LG9	25	Lu3083_323	39.289	Cloutier et al. (2012)
LG9	26	Lu144b_0	39.591	Cloutier et al. (2009)
LG9	27	Lu3085_323	39.603	Cloutier et al. (2012)
LG9	28	Lu2809a_166	41.881	Cloutier et al. (2012)
LG9	29	Lu2361_42*	42.403	Cloutier et al. (2012)
LG9	30	Lu2449_77*	42.953	Cloutier et al. (2012)
LG9	31	Lu3199a_444*	47.73	Cloutier et al. (2012)
LG9	32	Lu2828_175	52.113	Cloutier et al. (2012)
LG9	33	Lu2824_175	52.114	Cloutier et al. (2012)
LG9	34	Lu757_230	56.227	Cloutier et al. (2009)
LG9	35	Lu798_230*	56.243	Cloutier et al. (2009)
LG9	36	Lu2939a_230*	56.544	Cloutier et al. (2012)
LG9	37	Lu895_0*	61.469	Cloutier et al. (2012)
LG9	38	Lu2168_9	63.968	Cloutier et al. (2012)
LG9	39	Lu2878_196	64.949	Cloutier et al. (2012)
LG9	40	Lu1146b_196	69.992	Cloutier et al. (2012)
LG9	41	Lu2538_79*	85.779	Cloutier et al. (2012)
LG9	42	Lu3244_592*	87.491	Cloutier et al. (2012)
LG9	43	Lu932_490	90.606	Cloutier et al. (2012)
LG9	44	Lu1125_841	90.613	Cloutier et al. (2012)
LG9	45	Lu283_841	92.004	Cloutier et al. (2009)
LG9	46	Lu3216_490	93.411	Cloutier et al. (2012)
LG9	47	Lu91_490	94.627	Cloutier et al. (2009)

	S/n	Locus	Position	
LG10	1	Lu2472_71*	0	Cloutier et al. (2012)
LG10	2	Lu1158_71	2.124	Cloutier et al. (2012)
LG10	3	Lu668_71*	3.676	Cloutier et al. (2009)
LG10	4	Lu2149_5*	10.022	Cloutier et al. (2012)
LG10	5	Lu685_5*	12.929	Cloutier et al. (2009)
LG10	6	Lu2155_5	15.106	Cloutier et al. (2012)
LG10	7	Lu2157_5*	17.164	Cloutier et al. (2012)
LG10	8	Lu2154a_5	18.756	Cloutier et al. (2012)

LG10	9	Lu2162_7*	27.525	Cloutier et al. (2012)
LG10	10	Lu2901_204*	30.459	Cloutier et al. (2012)
LG10	11	Lu3099_353*	31.484	Cloutier et al. (2012)
LG10	12	Lu2052_32	31.508	Deng et al. (2010)
LG10	13	Lu3100b_353	31.748	Cloutier et al. (2012)
LG10	14	Lu273_32*	34.356	Cloutier et al. (2009)
LG10	15	Lu3007_269*	34.82	Cloutier et al. (2012)
LG10	16	Lu657_32*	34.901	Cloutier et al. (2009)
LG10	17	Lu1168_104*	35.757	Cloutier et al. (2012)
LG10	18	Lu3120_379*	36.429	Cloutier et al. (2012)
LG10	19	Lu2725_137	40.09	Cloutier et al. (2012)
LG10	20	Lu2728_137	42.109	Cloutier et al. (2012)
LG10	21	Lu2732_137*	42.476	Cloutier et al. (2012)
LG10	22	Lu458_137	42.732	Cloutier et al. (2009)
LG10	23	Lu2731_137*	45.981	Cloutier et al. (2012)
LG10	24	Lu2051_137*	47.102	Deng et al. (2010)
LG10	25	Lu2929_221*	52.987	Cloutier et al. (2012)
LG10	26	Lu2928_221*	54.964	Cloutier et al. (2012)
LG10	27	Lu2926_221	55.202	Cloutier et al. (2012)
LG10	28	Lu483_221*	56.005	Cloutier et al. (2009)
LG10	29	Lu37b_221*	58.624	Cloutier et al. (2009)
LG10	30	Lu1117_0*	59.371	Cloutier et al. (2012)
LG10	31	Lu1116_0*	59.63	Cloutier et al. (2012)
LG10	32	Lu1176_221*	60.561	Cloutier et al. (2012)
LG10	33	Lu2092b_0*	62.253	Roose-Amsaleg et al. (2006)
LG10	34	Lu2265_31*	64.921	Cloutier et al. (2012)
LG10	35	Lu2016_0	67.54	Deng et al. (2011)
LG10	36	Lu2050_0*	68.543	Deng et al. (2010)
LG10	37	Lu2264a_31*	69.664	Cloutier et al. (2012)
LG10	38	Lu2270_31	72.953	Cloutier et al. (2012)
LG10	39	Lu1043_780*	74.209	Cloutier et al. (2012)
LG10	40	Lu1136_31	74.438	Cloutier et al. (2012)
LG10	41	Lu1042_780*	75.401	Cloutier et al. (2012)
LG10	42	Lu2272_31*	76.231	Cloutier et al. (2012)
LG10	43	Lu371B_37*	81.749	Cloutier et al. (2009)
LG10	44	Lu2360_42	82.587	Cloutier et al. (2012)
LG10	45	Lu804_141*	84.63	Cloutier et al. (2009)
LG10	46	Lu2746_141	87.69	Cloutier et al. (2012)

	S/n	Locus	Position	
LG11	1	Lu2046_38*	0	Deng et al. (2010)
LG11	2	Lu2333_38	2.09	Cloutier et al. (2012)
LG11	3	Lu2332_38	3.455	Cloutier et al. (2012)
LG11	4	Lu2331_38	6.713	Cloutier et al. (2012)
LG11	5	Lu2867_188	9.477	Cloutier et al. (2012)
LG11	6	Lu11_0	10.613	Cloutier et al. (2009)
LG11	7	Lu2019_111	11.024	Deng et al. (2011)

LG11	8	Lu2676_111	13.49	Cloutier et al. (2012)
LG11	9	Lu2679a_111	20.72	Cloutier et al. (2012)
LG11	10	Lu2673_111	20.987	Cloutier et al. (2012)
LG11	11	Lu83_111	21.383	Cloutier et al. (2009)
LG11	12	Lu291_0	23.901	Cloutier et al. (2009)
LG11	13	Lu575_111	27.517	Cloutier et al. (2009)
LG11	14	Lu3218_497*	45.171	Cloutier et al. (2012)
LG11	15	Lu935_321*	48.626	Cloutier et al. (2012)
LG11	16	Lu3217_497	50.626	Cloutier et al. (2012)
LG11	17	Lu2850_187*	51.393	Cloutier et al. (2012)
LG11	18	Lu568_0	52.012	Cloutier et al. (2009)
LG11	19	Lu512_321*	55.101	Cloutier et al. (2009)
LG11	20	Lu785_321*	55.796	Cloutier et al. (2009)
LG11	21	Lu13_321*	57.945	Cloutier et al. (2009)
LG11	22	Lu3078_321	59.081	Cloutier et al. (2012)
LG11	23	Lu325_321*	60.126	Cloutier et al. (2009)
LG11	24	Lu934_0	61.101	Cloutier et al. (2012)
LG11	25	Lu292_0	61.195	Cloutier et al. (2009)
LG11	26	Lu2580_89*	69.1	Cloutier et al. (2012)
LG11	27	Lu3003_269*	71.491	Cloutier et al. (2012)
LG11	28	Lu2127b_2	82.152	Cloutier et al. (2012)
LG11	29	Lu2118_2*	84.243	Cloutier et al. (2012)
LG11	30	Lu2123_2*	85.599	Cloutier et al. (2012)
LG11	31	Lu1165_2*	86.202	Cloutier et al. (2012)

	S/n	Locus	Position	
LG12	1	Lu2917a_214*	0	Cloutier et al. (2012)
LG12	2	Lu628_78	6.522	Cloutier et al. (2009)
LG12	3	Lu14_0	8.032	Cloutier et al. (2009)
LG12	4	Lu927_78*	12.028	Cloutier et al. (2012)
LG12	5	Lu891_78	12.735	Cloutier et al. (2012)
LG12	6	Lu140_267	13.35	Cloutier et al. (2009)
LG12	7	Lu1041_0	13.417	Cloutier et al. (2012)
LG12	8	Lu756_267	15.133	Cloutier et al. (2009)
LG12	9	Lu2991_267	16.603	Cloutier et al. (2012)
LG12	10	Lu2041_0	17.995	Deng et al. (2010)
LG12	11	Lu2042_0	18.2	Deng et al. (2010)
LG12	12	Lu2694_115	18.689	Cloutier et al. (2012)
LG12	13	Lu2997_267	19.888	Cloutier et al. (2012)
LG12	14	Lu2992_36*	23.878	Cloutier et al. (2012)
LG12	15	Lu2996_267	27.031	Cloutier et al. (2012)
LG12	16	Lu220_267	27.334	Cloutier et al. (2009)
LG12	17	Lu2779_156*	29.998	Cloutier et al. (2012)
LG12	18	Lu2780_156*	30.642	Cloutier et al. (2012)
LG12	19	Lu2096_0	31.624	Roose-Amsaleg et al. (2006)
LG12	20	Lu867_0	31.731	Cloutier et al. (2012)
LG12	21	Lu926Bb_0	32.44	Cloutier et al. (2012)

LG12	22	Lu2774_156*	33.445	Cloutier et al. (2012)
LG12	23	Lu2880_197	34.655	Cloutier et al. (2012)
LG12	24	Lu2778_156*	36.966	Cloutier et al. (2012)
LG12	25	Lu2775a_156	37.657	Cloutier et al. (2012)
LG12	26	Lu2985_0	38.273	Cloutier et al. (2012)
LG12	27	Lu2773_156	39.665	Cloutier et al. (2012)
LG12	28	Lu2090_0	40.174	Roose-Amsaleg et al. (2006)
LG12	29	Lu2081_73*	40.501	Roose-Amsaleg et al. (2006)
LG12	30	Lu2482_73*	40.88	Cloutier et al. (2012)
LG12	31	Lu2486_73*	41.46	Cloutier et al. (2012)
LG12	32	Lu2600_97	41.719	Cloutier et al. (2012)
LG12	33	Lu2787_156*	41.856	Cloutier et al. (2012)
LG12	34	Lu2485_73*	42.426	Cloutier et al. (2012)
LG12	35	Lu2127a_2*	42.693	Cloutier et al. (2012)
LG12	36	Lu2183b_12*	43.024	Cloutier et al. (2012)
LG12	37	Lu2183c_12	43.275	Cloutier et al. (2012)
LG12	38	Lu1135_73	44.142	Cloutier et al. (2012)
LG12	39	Lu263_97*	44.489	Cloutier et al. (2009)
LG12	40	Lu896_0*	44.799	Cloutier et al. (2012)
LG12	41	Lu439_73	45.483	Cloutier et al. (2009)
LG12	42	Lu728a_161*	45.5	Cloutier et al. (2009)
LG12	43	Lu2612_97*	46.757	Cloutier et al. (2012)
LG12	44	Lu787b_156*	54.671	Cloutier et al. (2009)
LG12	45	fad3B_207	57.163	Cloutier et al. (2011)
LG12	46	Lu58b_257	58.82	Cloutier et al. (2009)
LG12	47	Lu2914_207	61.742	Cloutier et al. (2012)
LG12	48	Lu2913_207*	64.935	Cloutier et al. (2012)
LG12	49	Lu275_0*	68.6	Cloutier et al. (2009)
LG12	50	Lu206b_306	69.712	Cloutier et al. (2009)
LG12	51	Lu1052_0	70.014	Cloutier et al. (2012)
LG12	52	Lu203b_306	70.659	Cloutier et al. (2009)
LG12	53	Lu765Bb_306*	72.937	Cloutier et al. (2009)
LG12	54	Lu3063_306*	73.296	Cloutier et al. (2012)
LG12	55	Lu2574_84	75.34	Cloutier et al. (2012)
LG12	56	Lu2911_207	76.621	Cloutier et al. (2012)
LG12	57	Lu803_0	76.621	Cloutier et al. (2009)
LG12	58	Lu3064_306*	77.404	Cloutier et al. (2012)
LG12	59	Lu1151_0*	78.952	Cloutier et al. (2012)
LG12	60	Lu381_201	83.358	Cloutier et al. (2009)
LG12	61	Lu3289_1064	84.401	Cloutier et al. (2012)

	S/n	Locus	Position	
LG13	1	Lu2219_21	0	Cloutier et al. (2012)
LG13	2	Lu2223_21	11.819	Cloutier et al. (2012)
LG13	3	Lu2216_21	21.954	Cloutier et al. (2012)
LG13	4	Lu2074_0	25.895	Roose-Amsaleg et al. (2006)
LG13	5	Lu197_21	25.904	Cloutier et al. (2009)

LG13	6	Lu2468a_70	27.392	Cloutier et al. (2012)
LG13	7	Lu2639_103*	27.631	Cloutier et al. (2012)
LG13	8	Lu2638_103*	28.095	Cloutier et al. (2012)
LG13	9	Lu650_20*	28.938	Cloutier et al. (2009)
LG13	10	Lu2459_70	29.269	Cloutier et al. (2012)
LG13	11	Lu2467_70	29.291	Cloutier et al. (2012)
LG13	12	Lu2196_16	29.54	Cloutier et al. (2012)
LG13	13	Lu2463_70	30.796	Cloutier et al. (2012)
LG13	14	Lu485_0	32.612	Cloutier et al. (2009)
LG13	15	Lu2279_32	37.195	Cloutier et al. (2012)
LG13	16	Lu2771_154	42.091	Cloutier et al. (2012)
LG13	17	Lu805_11*	46.454	Cloutier et al. (2009)
LG13	18	Lu2468b_70	49.355	Cloutier et al. (2012)
LG13	19	Lu2176_11	65.977	Cloutier et al. (2012)
LG13	20	Lu2012_90	76.224	Deng et al. (2011)
LG13	21	Lu2021b_90	76.224	Deng et al. (2011)

	S/n	Locus	Position	
LG14	1	Lu2621_99	0	Cloutier et al. (2012)
LG14	2	Lu897_0	2.051	Cloutier et al. (2012)
LG14	3	Lu3251_630	5.025	Cloutier et al. (2012)
LG14	4	Lu3209_482	5.646	Cloutier et al. (2012)
LG14	5	Lu3210_482*	5.756	Cloutier et al. (2012)
LG14	6	Lu3212_482	6.085	Cloutier et al. (2012)
LG14	7	Lu2045_0	8.054	Deng et al. (2010)
LG14	8	Lu2373_45	12.975	Cloutier et al. (2012)
LG14	9	Lu2377_45	14.905	Cloutier et al. (2012)
LG14	10	Lu808_0	15.295	Cloutier et al. (2009)
LG14	11	Lu3043_290	17.159	Cloutier et al. (2012)
LG14	12	Lu3040_290	20.484	Cloutier et al. (2012)
LG14	13	Lu850_290	24.995	Cloutier et al. (2009)
LG14	14	Lu793_290	26.185	Cloutier et al. (2009)
LG14	15	Lu3033_290	26.706	Cloutier et al. (2012)
LG14	16	Lu514_290	26.851	Cloutier et al. (2009)
LG14	17	Lu3046_290	27.856	Cloutier et al. (2012)
LG14	18	Lu3219a_497	28.824	Cloutier et al. (2012)
LG14	19	Lu9_0	29.829	Cloutier et al. (2009)
LG14	20	Lu813_290	30.071	Cloutier et al. (2009)
LG14	21	Lu3038_290	32.156	Cloutier et al. (2012)
LG14	22	Lu3036_290	32.368	Cloutier et al. (2012)
LG14	23	Lu786_101	33.068	Cloutier et al. (2009)
LG14	24	Lu3103_355	33.396	Cloutier et al. (2012)
LG14	25	Lu225_355	40.335	Cloutier et al. (2009)
LG14	26	Lu2679b_111	41.011	Cloutier et al. (2012)
LG14	27	Lu601b_355	42.966	Cloutier et al. (2009)
LG14	28	Lu2625b_101	43.563	Cloutier et al. (2012)
LG14	29	Lu444_188	47.145	Cloutier et al. (2009)

LG14	30	Lu476_188	47.931	Cloutier et al. (2009)
LG14	31	Lu461_188	48.3	Cloutier et al. (2009)
LG14	32	Lu2863_188	50.968	Cloutier et al. (2012)
LG14	33	Lu684_188	51.556	Cloutier et al. (2009)
LG14	34	Lu2865_188	53.046	Cloutier et al. (2012)
LG14	35	Lu613_188	53.252	Cloutier et al. (2009)
LG14	36	s7F06_0	56.704	Cloutier et al. (2011)
LG14	37	Lu2862_188	61.585	Cloutier et al. (2012)
LG14	38	Lu2020_89	64.128	Deng et al. (2011)
LG14	39	s16E2_0	67.799	Cloutier et al. (2011)
LG14	40	s19C1c3_0	68.515	Cloutier et al. (2011)
LG14	41	Lu701_89	68.653	Cloutier et al. (2009)
LG14	42	Lu1044_89	70.086	Cloutier et al. (2012)
LG14	43	Lu1044B_89	70.177	Cloutier et al. (2012)
LG14	44	Lu959_89	70.786	Cloutier et al. (2012)
LG14	45	s19C1c1_0	76.015	Cloutier et al. (2011)

	S/n	Locus	Position	
LG15	1	Lu2497_74	0	Cloutier et al. (2012)
LG15	2	Lu451_0	1.465	Cloutier et al. (2009)
LG15	3	Lu1001_74	1.967	Cloutier et al. (2012)
LG15	4	Lu637_226	2.679	Cloutier et al. (2009)
LG15	5	Lu271_226*	6.321	Cloutier et al. (2009)
LG15	6	Lu2931_226*	7.073	Cloutier et al. (2012)
LG15	7	Lu510_440	8.718	Cloutier et al. (2009)
LG15	8	Lu3186_440*	11.916	Cloutier et al. (2012)
LG15	9	Lu3026_287*	13.542	Cloutier et al. (2012)
LG15	10	Lu3185_440	14.743	Cloutier et al. (2012)
LG15	11	Lu3028_287	17.273	Cloutier et al. (2012)
LG15	12	Lu2707_121*	19.225	Cloutier et al. (2012)
LG15	13	Lu2383_50*	20.87	Cloutier et al. (2012)
LG15	14	Lu1163_121	22.241	Cloutier et al. (2012)
LG15	15	Lu2057_692*	23.059	Deng et al. (2010)
LG15	16	Lu357_42	23.948	Cloutier et al. (2009)
LG15	17	Lu2382_50	24.051	Cloutier et al. (2012)
LG15	18	Lu2965_243*	24.489	Cloutier et al. (2012)
LG15	19	Lu1127_121	24.991	Cloutier et al. (2012)
LG15	20	Lu2001_0*	25.588	Deng et al. (2011)
LG15	21	Lu2010a_190	28.05	Deng et al. (2011)
LG15	22	Lu359_0*	41.977	Cloutier et al. (2009)
LG15	23	Lu2354_40*	42.829	Cloutier et al. (2012)
LG15	24	Lu1007_40	46.393	Cloutier et al. (2012)
LG15	25	Lu1172_118	48.197	Cloutier et al. (2012)
LG15	26	Lu838_212*	48.921	Cloutier et al. (2009)
LG15	27	Lu462a_212*	48.986	Cloutier et al. (2009)
LG15	28	Lu2696_118*	52.104	Cloutier et al. (2012)
LG15	29	Lu2695_118	52.458	Cloutier et al. (2012)

LG15	30	Lu113_118	53.254	Cloutier et al. (2009)
LG15	31	fad2B_405	57.836	Cloutier et al. (2011)
LG15	32	Lu2697b_118*	59.903	Cloutier et al. (2012)
