# Supplementary material for: Integrated consensus genetic and physical maps of flax (Linum usitatissimum L.)
Source: Theor Appl Genet. 2012 Aug 14;125(8):1783–95. doi: 10.1007/s00122-012-1953-0 (PMC3493668; doi:10.1007/s00122-012-1953-0)
Supplement: Supplementary file 3 — Supplementary material 3 (PDF 38 kb) [file 122_2012_1953_MOESM3_ESM.pdf]

Table S2 Distribution of loci and distorted loci across the linkage groups of the three individual maps and the consensus map

Linkage groups	CDC Bethune/Macbeth (BM)				E1747/Viking (EV)				SP2047/UGG5-5 (SU)				Consensus	
	No of loci	Distorted loci	Distorted loci		No of loci	Distorted loci	Distorted loci		No of loci	Distorted loci	Distorted loci		No of loci	Distorted loci
			B	M			E	V			S	U		
LG1	23	4	4	0	46	2	1	1	37	16	16	0	64	22
LG2	38	10	1	9	36	7	0	7	46	19	4	15	65	36
LG3	35	4	4	0	32	10	10	0	44	3	1	2	64	17
LG4	36	3	2	1	30	3	3	0	32	14	5	9	53	20
LG5	23	0	0	0	21	1	1	0	43	2	0	2	62	3
LG6	40	12	3	9	38	3	2	1	25	10	8	2	59	25
LG7	24	4	4	0	25	0	0	0	38	6	0	6	52	10
LG8	37	3	0	3	39	6	6	0	47	43	43	0	68	52
LG9	22	1	1	0	19	1	0	1	26	15	1	14	47	17
LG10	17	4	3	1	30	20	0	20	31	8	8	0	46	32
LG11	12	0	0	0	21	1	1	0	20	12	12	0	31	13
LG12	31	1	0	1	33	8	1	7	32	16	2	14	61	25

LG13	12	4	2	2	14	0	0	0	1	0	0	0	21	4
LG14	16	0	0	0	21	1	1	0	24	0	0	0	45	1
LG15	10	2	0	2	27	9	2	7	23	4	4	0	32	15
LG16	9	4	4	0	5	5	5	0						
LG17					2	0	0	0						
LG18					3	0	0	0						
Total	385	56	28	28	442	77	33	44	469	168	104	64	770	292
Percent		14.5	50.0	50.0		17.4	42.9	57.1		35.8	61.9	38.1		37.9
