# Supplementary material for: Integrated consensus genetic and physical maps of flax (Linum usitatissimum L.)
Source: Theor Appl Genet. 2012 Aug 14;125(8):1783–95. doi: 10.1007/s00122-012-1953-0 (PMC3493668; doi:10.1007/s00122-012-1953-0)
Supplement: Supplementary file 4 — Supplementary material 4 (PDF 15 kb) [file 122_2012_1953_MOESM4_ESM.pdf]

Table S3 Common loci integrated in each linkage group of the consensus map

Linkage groups	No. loci in consensus map	Common loci			
		BM/EV	BM/SU	EV/SU	BM/EV/SU
LG1	64	2	3	16	10
LG2	65	3	8	10	17
LG3	64	1	8	12	13
LG4	53	4	12	5	12
LG5	62	5	5	3	3
LG6	59	13	8	3	10
LG7	52	3	7	13	6
LG8	68	5	5	11	17
LG9	47	3	5	6	3
LG10	46	2	5	14	5
LG11	31	2	0	7	3
LG12	61	4	4	7	9
LG13	21	4	0	0	1
LG14	45	3	3	5	2
LG15	32	6	1	11	3
Total	770	60	74	123	114
